# Rapid Reconstruction of 3D Neuronal Morphology from Light Microscopy Images with Augmented Rayburst Sampling

**DOI:** 10.1371/journal.pone.0084557

**Published:** 2013-12-31

**Authors:** Xing Ming, Anan Li, Jingpeng Wu, Cheng Yan, Wenxiang Ding, Hui Gong, Shaoqun Zeng, Qian Liu

**Affiliations:** 1 Britton Chance Center for Biomedical Photonics, Huazhong University of Science and Technology - Wuhan National Laboratory for Optoelectronics, Wuhan, China; 2 MoE Key Laboratory of Biomedical Photonics, Department of Biomedical Engineering, Huazhong University of Science and Technology, Wuhan, China; SUNY Downstate MC, United States of America

## Abstract

Digital reconstruction of three-dimensional (3D) neuronal morphology from light microscopy images provides a powerful technique for analysis of neural circuits. It is time-consuming to manually perform this process. Thus, efficient computer-assisted approaches are preferable. In this paper, we present an innovative method for the tracing and reconstruction of 3D neuronal morphology from light microscopy images. The method uses a prediction and refinement strategy that is based on exploration of local neuron structural features. We extended the rayburst sampling algorithm to a marching fashion, which starts from a single or a few seed points and marches recursively forward along neurite branches to trace and reconstruct the whole tree-like structure. A local radius-related but size-independent hemispherical sampling was used to predict the neurite centerline and detect branches. Iterative rayburst sampling was performed in the orthogonal plane, to refine the centerline location and to estimate the local radius. We implemented the method in a cooperative 3D interactive visualization-assisted system named flNeuronTool. The source code in C++ and the binaries are freely available at http://sourceforge.net/projects/flneurontool/. We validated and evaluated the proposed method using synthetic data and real datasets from the Digital Reconstruction of Axonal and Dendritic Morphology (DIADEM) challenge. Then, flNeuronTool was applied to mouse brain images acquired with the Micro-Optical Sectioning Tomography (MOST) system, to reconstruct single neurons and local neural circuits. The results showed that the system achieves a reasonable balance between fast speed and acceptable accuracy, which is promising for interactive applications in neuronal image analysis.

## Introduction

Digital reconstruction of neuronal morphology from light microscopy images provides a powerful technique for the analysis of neural circuits forms and for the investigation of their underlying function [1]. Since the manual reconstruction is very time-consuming, especially for large-scale neuronal analysis, a number of studies have been conducted to develop more efficient computer-assisted approaches to support neurite tracing and neuronal morphology reconstruction [2]–[4].

Methods of neurite tracing roughly fall in three categories. The first category relies on the sequential presentation of serial 2D images. These methods extract the profiles of neural structures in each 2D cross-sectional plane and then connect the results in the third dimension [5]–[7]. However, this may be in trouble when local neurite segments lie parallel to the 2D plane. Resampling the original images along different directions might work but is computationally expensive and difficult to cover all neurites.

The second category is based on global image segmentation in 3D. These methods first turn the image into a binary form with certain segmentation algorithms and then extract centerlines of foreground areas with a skeletonization algorithm [8], [9]. Here, efficient segmentation algorithms, such as thresholding, prove successful for uniformly high quality images, although it is not always the case. Various sophisticated filters have been proposed to enhance feature structures and to improve segmentation and skeletonization. Typically, such filters are based on the analysis of eigenvalues of the Hessian [10], Jacobian matrix [11], or steerable filters [12]. However, these filters require multiple scaling or orientations and are computationally expensive because they operate on the entire image. A few other algorithms, such as voxel coding [13], [14] and voxel scooping [15], can trace multiple neurite branches directly from the grayscale images but they operate on a voxel-by-voxel basis and are more dependent on the high quality of pre-processed images.

The third category explores images only in local regions around the structures of interest. These methods usually start from a seed, which can be located automatically or manually, and trace recursively where neurite branches go in the 3D image. The direction of tracing is determined according to the distribution of local signal and background. A representative algorithm is to use a template that comprises four predefined parallel edge detectors, to determine the direction and boundary of a single branch [16]–[18]. More robust algorithms often rely on certain mathematical or graph models. They usually define a cost or energy measure that is based on local image features and curve regularity; then convert the problem into searching for an optimal path or minimum spanning tree between given crucial points [19]–[25]. If initial structures or prior knowledge can be obtained, it then becomes optional to fit a snake-based or active contour model to the image data [26]–[28]. A potential problem is that these local tracing algorithms may fail at branch points or crossover regions because of a lack of global information. Thus, a separate branch merger or segment join step is needed to trace whole tree-like structure [29]–[31].

To reconstruct 3D neuronal morphology, the neurite tracing can be coupled with radius estimation at each node. The rayburst sampling proposed by Wearne and Rodriguez can be used for this [32], [33]. It samples the image data in multiple directions using a pre-computed array of unit vectors from a given node. The process of sampling is defined as the simultaneous casting of rays in multiple directions. Each ray grows from a given node until a specified exit criterion is met, which returns a length representing the forward sampling distance. In the original algorithm, a 2D rayburst is run in the cross-sectional plane for each node, and the diameter is computed from the ray lengths. It is worth noticing that to reconstruct the whole dendritic tree, it is necessary to extract the centerline of branches in advance.

The methods mentioned above are all helpful for the reconstruction of neuronal morphology from light microscopy images. However, these automated reconstructions often contain a substantial number of false segments or short branches, requiring post-correction with significant human effort [34], [35]. Thus, to achieve a reasonable balance between fast speed and high accuracy, a rapid reconstruction system incorporating automatic tracing and manual editing is preferable.

In this paper, we present a practical method for the tracing and reconstruction of 3D neuronal morphology from light microscopy images and implement it in an interactive visualization-assisted freeware system named flNeuronTool. We extend the original rayburst sampling algorithm to a marching fashion, which starts from a single or a few initial seed points and marches recursively forward along neurite branches to trace and perform reconstruction on the whole tree-like structure. We validate and evaluate the method using synthetic data and real datasets from the Digital Reconstruction of Axonal and Dendritic Morphology (DIADEM) challenge [36], [37]. Then, the system is applied to mouse brain images acquired with the Micro-Optical Sectioning Tomography (MOST) system [38]–[41], to reconstruct single neurons and local neural circuits.

## Methods

### Neurite Tracing Strategy

At the typical resolution of light microscope images, each segment of a neurite has a smooth tubular shape, which can be approximated as a generic cylinder with a certain radius [42]. Moreover, the diameter and direction of a neurite in a local area never changes abruptly, which means that two adjacent segments have a similar radius and their centerlines often form a modest angle. Thus, it is possible to predict the radius and direction of the next segment according to the current presentation [43].

**Figure 1:**
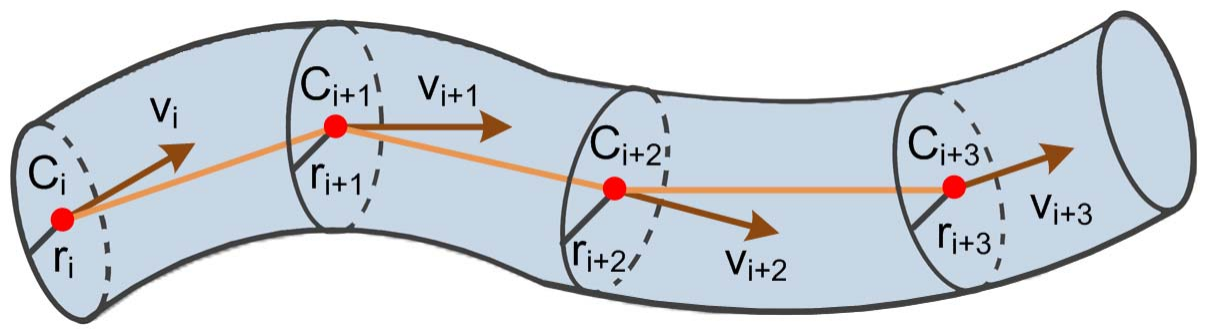
Neurite tracing strategy. A node is recursively moved along the centerline of a neurite, to reconstruct neurite segments as a sequence of cylinders. At any given position, the node has a location C, a direction v and a radius r.

We trace individual neurite using a prediction and refinement strategy. As shown in **Figure 1**, we recursively move a node along the centerline of a neurite. For any given position, the node has three properties: the location C is a point on the centerline; the direction v is a unit vector that represents the tangent of the centerline; and the radius r is the minimum distance from point C to the structure boundary, which is estimated at the orthogonal plane of the direction v. Two consecutive nodes are defined as the parent and child nodes, respectively. If appropriate properties of the parent node have been obtained, we can predict the child node position along direction v. Then, using local images, the properties of the child node can be refined. Recursively, we can trace the whole tree starting from one single or a few of seed points.

### Centerline Extraction and Branch Detection

**Figure 2:**
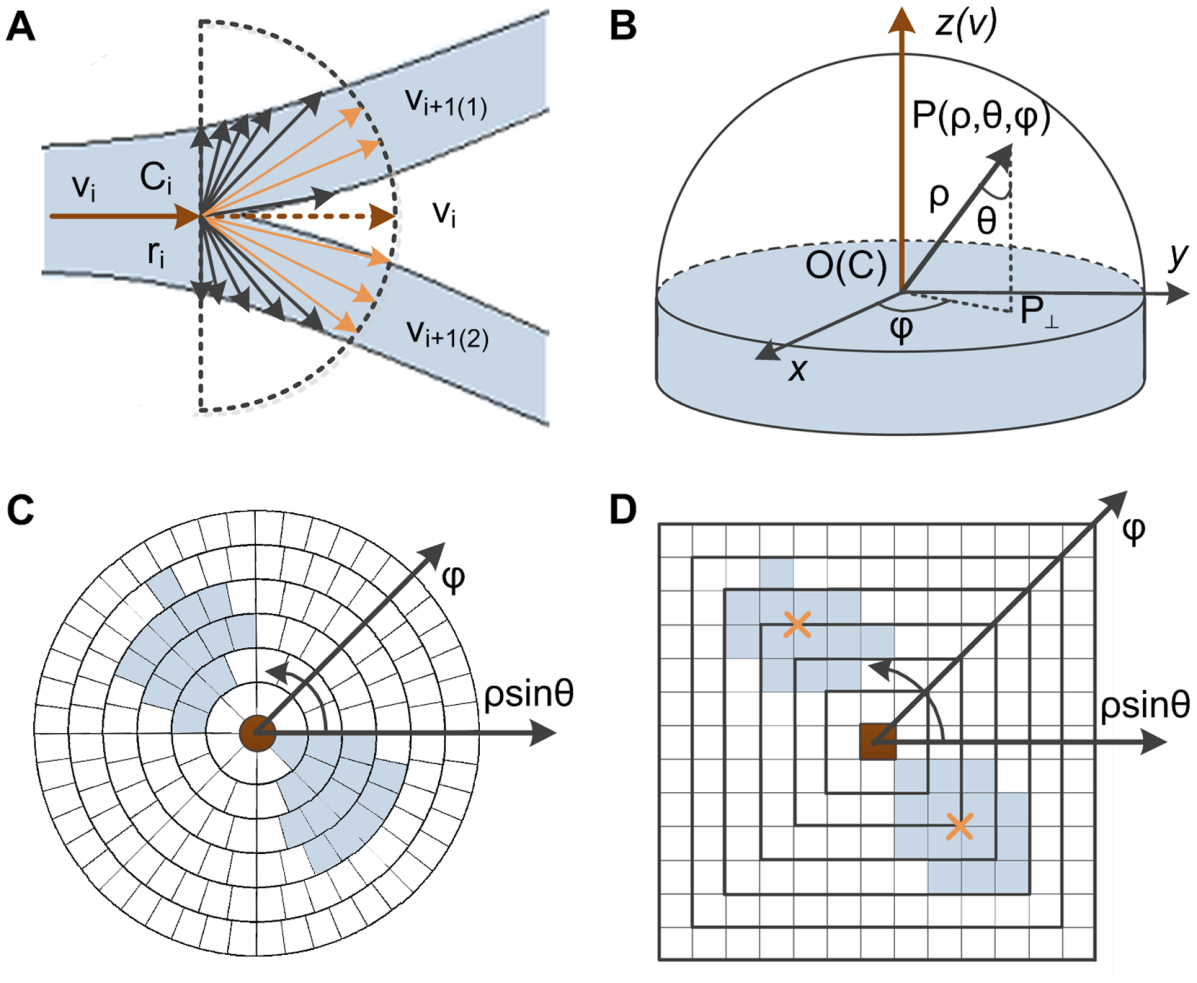
Centerline extraction and branch detection using hemisphere sampling. (A) 2D illustration of the centerline and branch prediction with improved rayburst sampling. (B) 3D illustration of a vector of the hemisphere sampling core in a spherical coordinate system. (C) The sampling state on the hemispherical surface is projected to the orthogonal plane, in which the sampling rays that have the same inclination angle θ form a circle, and nonzero value samplings (bright blue) suggest possible branches. (D) In the plane, each circle can be mapped into a circumscribed square, and each local center detected (orange) represents a neurite branch.

We use augmented rayburst sampling to detect centerlines and branches in the prediction step. Inspecting 2D rayburst sampling at a given node, we find that the longest rays are always close to the direction of the axis that represents the potential centerline of the local neurite segment, while the shortest rays in the orthogonal direction represent the local radius. Consequently, when extending all of the rays from the position of the current node until their length goes over a specified threshold (approximately 2.0∼4.0 times the local radius), those rays that are far away from the axis will reach or exceed the boundary of the neurite, and the corresponding samplings will been terminated. The remaining rays that are still inside the structure will be separated into a few clusters according to their directions, which represent potential branches. Specifically, they are together as a single cluster when the branches are absent. The length threshold of each ray is defined as the sampling distance, which is only related to the local radius but independent of the absolute size of the structural structure. Moreover, to avoid backward tracing, we perform sampling only in the forward direction. As shown in **Figure 2A**, actual sampling could be restricted to a half circle, whose origin locates at the current node position C, and the half angle ray represents the node direction v.

The improved rayburst sampling is introduced for 3D. Here, to detect branches, sampling is restricted to a hemisphere; thus, it is crucial to generate an approximately uniformly distributed sampling core on the hemisphere surface. The random generation or polygon subdivision used in the original algorithm cannot achieve this goal. As shown in **Figure 2B**, we solve this problem in a spherical coordinate system for which the origin O is located at the current node position C, the z axis coincides with the current node direction v and the orthogonal plane parallels the cross-section that covers the local radius r. For any vector OP with length ρ, its endpoint coordinate is P(ρ, θ, φ), in which θ and φ represent the inclination and azimuthal angles, respectively. We divide the whole hemisphere into M (M = 7) stacks according to the inclination angle θ, with equal intervals. For each given θ, we divide corresponding small circles into N slices according to the azimuthal angle φ, with equal intervals. The angular distribution is expressed as
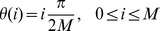


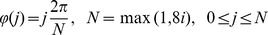
where the function max selects the maximum value from two given numbers, and the reason for setting the variable N values will be explained below. To sample the original data, vector OP should be converted into the Cartesian form, in which the coordinate of the endpoint P(x, y, z) is expressed as



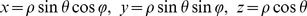



It is convenient to set ρ to one for automated normalization. Nevertheless, in some case, where neurite segments are really thin and comparatively straight, one can speed up the sampling in the z direction. Thus, an anisotropic sampling core is optional and has no influence on the sampling form, while the conversion to the z axis is expressed as
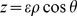
where ε is defined as the anisotropy coefficient (approximately 1.0∼1.1). Then, these vectors could be normalized and used as a per-computed sampling core.

To perform 3D hemispherical sampling at a given node, we rotate the sampling core to correct the orientation using a transformation matrix, by which we match the current node direction v with the z axis. Then, each vector in the sampling core is iteratively extended with a pre-specified step-length from the current position C until the length goes over the pre-specified sampling distance. Those rays who reach the boundary of the local structure should be terminated early. One could use an adaptive hysteresis threshold to detect the boundary along a sampling ray (supporting information, **Text S1** in **File S1**). In the end of the sampling setup, the sampling state whose ray endpoint is still in the inside of the structure is recorded as a value of one, and the remaining is recorded as a value of zero. A possible set of sampling state on the hemispherical surface is projected to the orthogonal plane as shown in **Figure 2C**, in which the sampling rays that have the same inclination angle θ form a circle, and nonzero value samplings suggest possible branches.

In the plane, each circle can be mapped into a circumscribed square with slight deformation, as shown in **Figure 2D**. Consequently, these sampling squares with different inclination angles θ and azimuthal angles φ form a uniform grid. Besides, the number of samplings in the innermost square is only one, and the difference between the adjacent squares is 8, which is the reason for setting the variable N values for the sampling core generation, as described above. In this uniform grid, nonzero value samplings are separated into a few areas for which the local centers could be detected by multiform ways, such as a simple and effective distance transform [44]. In general, each local center represents a potential neurite branch that can be mapped back to the hemisphere surface and is expressed in both spherical coordinates and the Cartesian form. Then, a new node can be located in the branch as the child whose parent is the current node. The distance between it and the parent node is defined as the location distance, which is approximately equal to a diameter of the parent node.

### Centerline Refinement and Radius Estimation

The location of a node that is predicted by hemispherical sampling may be a bit off the real centerline of local neurite because of holes or other artifacts in light microscopy images, and the radius of predicted child node equals the parent node radius, which should be refined. We run an improved 2D rayburst sampling to perform this step.

**Figure 3:**
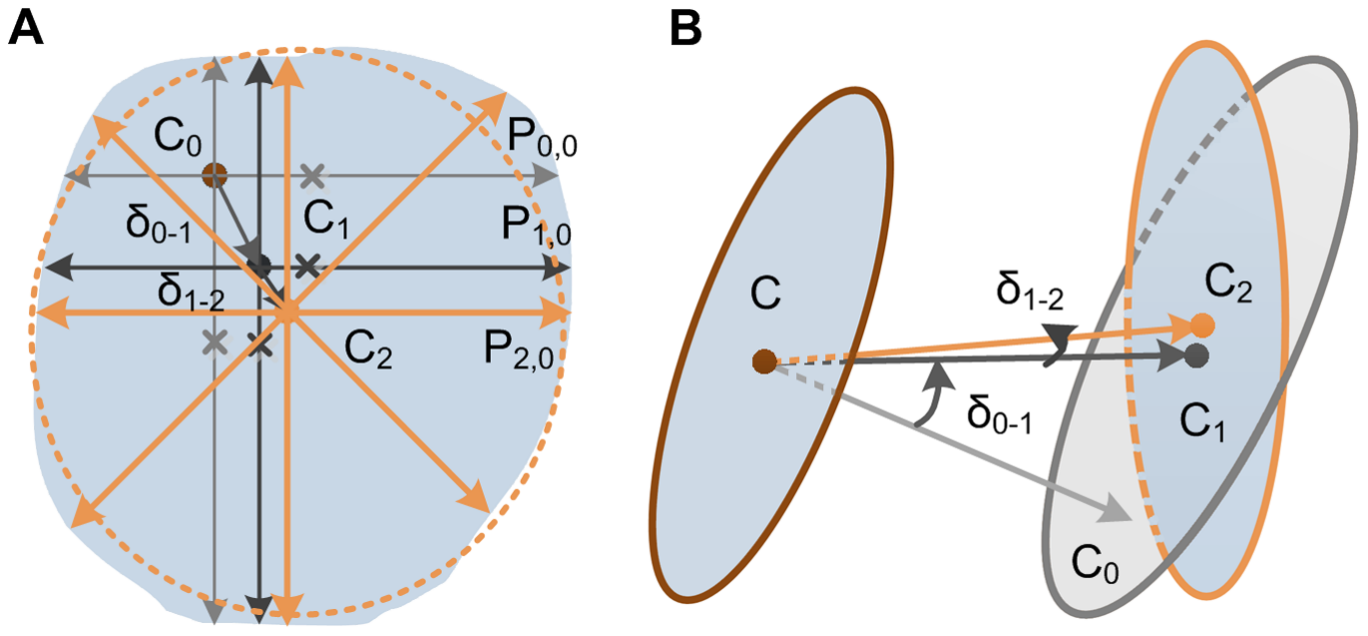
Centerline refinement and local radius estimation using iterative rayburst sampling. (A) Centroid refinement and local radius estimation using iterative 2D rayburst samplings in which each calculated centroid is regarded as the origin for the next sampling. (B) 3D illustration of centerline refinement. The sampling described above is iterated in a plane that is orthogonal to the current centerline direction.

As shown in **Figure 3A**, a set of unit vectors that are parallel to the sampling plane and uniformly distributed in a circle are used as a sampling core. The sampling starts at the predicted node C. Each ray iteratively extends with a pre-specified step-length from the origin point until the structure boundary is reached. We record the intersection of a ray and the boundary as P. Ideally, the cross-section is a generic ellipse, of which a centroid is simply the average location of all intersections. For real images, to gradually approach to the real position, we iteratively perform multiple sampling in which each calculated centroid is regarded as the origin for the next sampling. This process is terminated when the Euclidean distance δ between two calculated positions from successive iterations is less than a pre-specified threshold, which is a radius-related value (the coefficient ε = 0.05). For the last iteration, the lengths of all of the rays are also calculated, of which the mean is regarded to be the estimated radius r of the current node. This process is expressed as
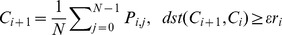


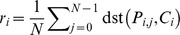
where N (N = 32) is the number of the 2D sampling rays, P_i, j_ represents the intersection position of the ray numbered j and the boundary in the iteration numbered i, and the function dst calculates the Euclidean distance between two given points.

The segment from the parent node to the current node represents the local centerline. However, a potential problem is that it could no longer be orthogonal to the reconstructed circle because the sampling origin used to refine the centerline and estimate the radius has shifted during the iterations. There is a space angle δ between the initial direction that was predicted by hemispherical sampling and the refined direction, which suggests a calculation error of the centerline, as shown in **Figure 3B**. To improve the precision of the centerline and radius refinements, we iterate the step described above in a new plane that is orthogonal to the current direction until the angle between the last direction and the prior direction falls under a pre-specified threshold (δ = π/36), which result in an appropriate precision while preventing unnecessary iteration. Then, the cross-section of neurite is reconstructed as a circle with a radius r that locates at the position C and is approximately orthogonal to the refined centerline.

### Reconstruction of Neuronal Morphology

The algorithm described above is implemented in a freeware system named flNeuronTool, which is programmed in C++ and potentially supports multiple platforms. This system allows users to reconstruct and proofread neuronal morphologies in a cooperative 3D interactive visualization-assisted environment (supporting information, **Text S2** in **File S1**). The source code and binaries are freely available at http://sourceforge.net/projects/flneurontool/.

To run the tracing algorithm, an initial node has to be created as the seed point. We select one single or a few points that are inside of a neuron and are close to the root of the tree-like structure, and pushed them into a queue as seed points. This can be automatically done, but we do prefer a manual way in a 3D interactive environment, which can significantly reduce false positive errors. At each selected location, two hemisphere samplings that have opposite directions are carried out to detection branches. The rest of tracing process is recursive prediction and refinement as described above. In every recursion, generated nodes are all pushed in the queue, which will be orderly popped as new seed points. When all seed points are run out and the sampling end, one could select a new seed point to reconstruct remaining branches of the tree-like structure. This process iterates until the expected reconstruction is achieved.

Due to imperfect images or excessive seed points, the reconstruction will have a number of redundancy paths and short branches. Here, redundancy paths cover the same neurite, in which each pair corresponding nodes have a similar direction and radius, and cover a more or less overlap area. One could find and merge all such node pairs from the root to endpoints based on whether the Cartesian distance between them is less than any one of them radius [23]. The centroid of each pair nodes is calculated to produce a final node location, while radius is averaged. As additional gains, this method uses redundancy paths to generate a more complete and better reconstruction. The length of a branch is defined as the path length that from the last bifurcation to its endpoint. One could remove short branches based on their absolute length and their lengths relative to the local radius [15].

## Experiments and Results

### Parameters Selection

To validate and evaluate the proposed method, we tested it on synthetic data and real datasets from the DIADEM challenge. Then, we applied the system to mouse brain images acquired with the MOST system to reconstruct single neurons and local neural circuits. All of the experiments were performed on an ordinary computer (Intel Core Duo 2.6 GHz CPU, NVIDIA GeForce 9800 GPU, 4GB RAM, Windows 7). **Table 1** summarizes the sampling parameters for these experiments, in which most parameters remain constant across all experiments, while other parameters need to be adapted for each dataset to produce optimal results.

**Table 1:** Sampling parameters selection.

Parameters	Value	Notes
3D sampling distance	2.0∼4.0 (radius)	Each ray is terminated when its length is over the threshold. A value too big or too small will lead to the branch detection lost or redundancy. Default value is 3.0.
Anisotropy coefficient	1.0∼1.1	An anisotropic sampling core can speed up the sampling in the axial direction. Default value is 1.0.
Inclination division number	7	This value determines the number of 3D sampling rays. The larger value will create a more delicate sampling core, but will require more calculated amount.
Sampling step-length	1.0 (voxel)	Each ray is iteratively extending with the step-length from the origin until the exit criterion is satisfied. For anisotropic images, a super sampling should be applied in the low resolution direction.
Location distance	1.5∼2.5 (radius)	The distance between the child and the parent node affects the density of nodes in the reconstruction. Default value is 2.0 but no more than the 3D sampling distance.
2D sampling ray number	32	These rays are used to refine centerline and radius.
Iteration exit distance	0.05 (radius)	Centroid refinement is terminated when the distance between two successive locations is less than the threshold.
Iteration exit angle	π/36	Centerline refinement is terminated when the angle between two successive directions is less than the threshold.

### Validation on Synthetic Data

**Figure 4:**
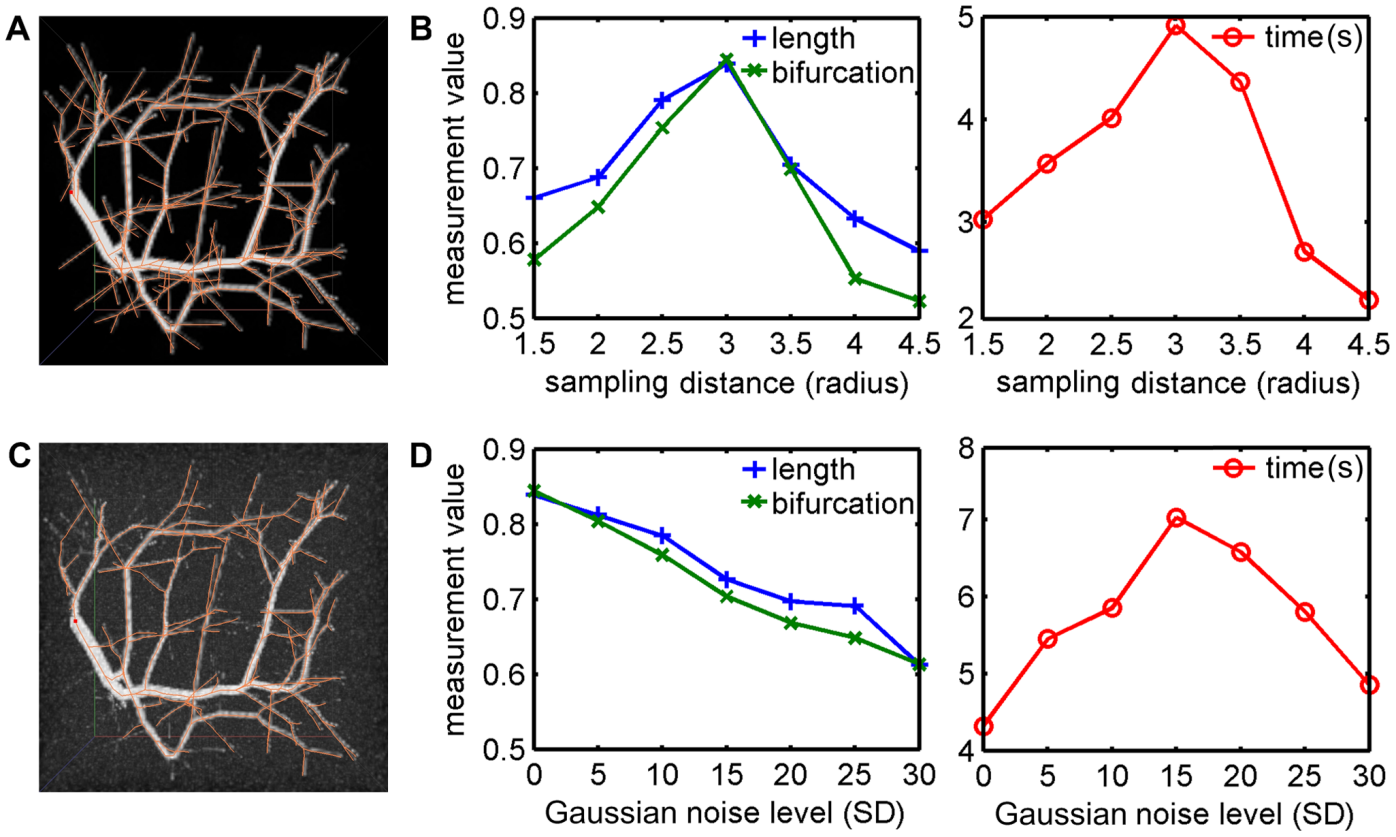
Validation of the branch detection on tree-like structure data. (A) The original image (voxel size 101×101×101) and truth structure (branch length 3325 voxels, bifurcation number 199). (B) The performance analysis for the tracing of the tree structure in the original image with different sampling distance. For each tracing, only one seed point is used. The total length of correct branches and the number of correct bifurcations are measured, and the proportions of them to truth values are reported, as well as the computation time in seconds. (C) The highest Gaussian noised image (SD 30) and the corresponding automated reconstruction (branch length 2036 voxels, bifurcation number 122). (D) The robustness analysis for the tracing of the tree structure in different level Gauss noised images with the same sampling distance (3.0 times local radius).

First, we inspected the performance of the branch detection using synthetic tree-like structure data. The original image and the corresponding truth structure were created by VascuSynth [45] and were downloaded from website (http://vascusynth.cs.sfu.ca/), as shown in **Figure 4A**. To find the optimal value of the 3D sampling distance for the branch detection, we traced the tree in the image with different sampling distance, which ranges from 1.5 to 4.5 with an increment of 0.5 times the local radius (supporting information, **Text S3** in **File S1**). For each tracing, we measured the total length of correct branches and the number of correct bifurcations, and reported the proportions of them to true values. Because the test was run directly on the image without any additional pre-processing or manual editing and used only one seed point that was located at the root of the tree by one mouse click, we could report the computation time for each tracing, as shown in **Figure 4B**. From the trend in these measurements, it is found that a sampling distance too big or too small would lead to the branch detection lost or redundancy, and the optimal value is about 3.0. Considering the difference of images, one may set this value between 2.0 and 4.0. In general, the value of length is always more than the value of the bifurcations and has smaller change, for which lost branches are relatively short.

Then, we validated the robustness of the branch detection. We used ImageJ (http://rsb.info.nih.gov/ij/) to generate a set of images based on the original tree-like structure data with additive Gaussian noise, whose standard deviation (SD) ranges from 5 to 30 with an increment of 5. **Figure 4C** shows the highest noised image and the corresponding automated reconstruction. For each image, the value of the sampling distance was set to 3.0. Once again, we reported the length of branches, the number of bifurcations and the computation time, as shown in **Figure 4D**. It is obvious that the tree-like structure becomes more ambiguous when the noise is higher, making the tracing more difficult. However, considering that there is only one seed point for each tracing, the result is acceptable. Overall, low-level noise could lead to more short branches, which must be removed expending additional time. High-level noise tends to cause the early termination of tracing, which is helpful for reducing false positive errors.

### Evaluation with DIADEM Dataset

We evaluated the proposed method with two real datasets from the DIADEM challenge. Specifically, the two datasets were drosophila olfactory projection (OP) [46] and mouse neuromuscular projection fibers (NM) [47]. The image stacks and their gold standard reconstructions were downloaded from website (http://www.diademchallenge.org/).

**Figure 5:**
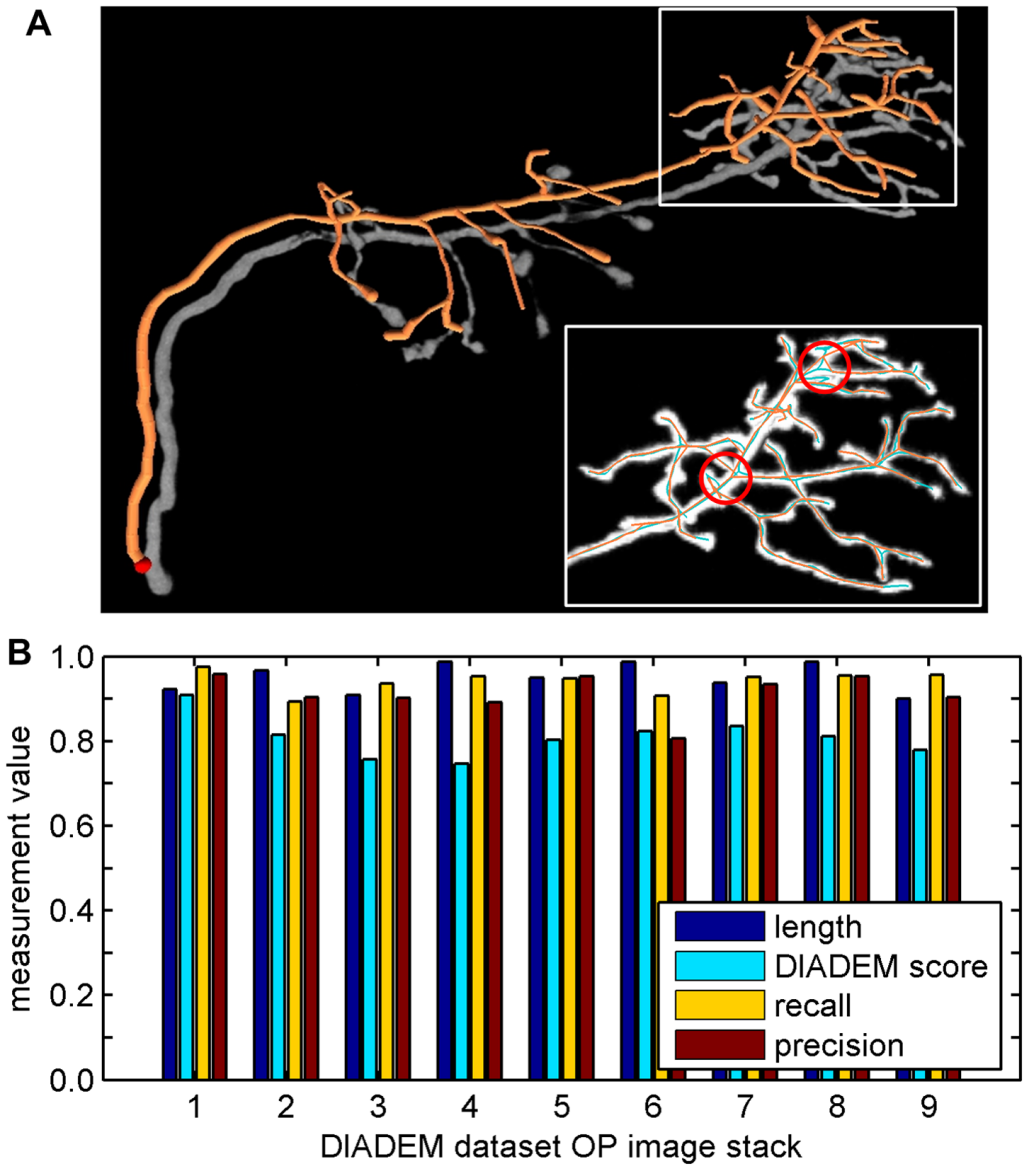
Evaluation with the DIADEM dataset OP. (A) The original data and the automated reconstruction of the first image stack (voxel size 512×512×60), which is shifted slightly relative to the voxel data to improve the visibility. Local detail of the reconstruction (orange) and gold standard (cyan) are displayed in the inset, in which potential errors are circled (red). (B) The quantitative measurements of nine image stacks. The DIADEM score, recall and precision are report based on DIADEM metric measurements compare with the gold standards. Specifically, the path length and DIADEM score are approximately 0.95±0.03 (mean ± SD) and 0.81±0.05.

The first test was run on 9 image stacks of dataset OP. For each image stack, we traced three times with different sampling distances that range from 2.5 to 3.5, from which the one that had longest path was selected as final reconstruction. The final reconstruction of the first stack is shown in **Figure 5A**, in which the reconstruction is shifted slightly relative to the voxel data to improve the visibility. Local detail of the reconstruction and the corresponding gold standard are displayed in different colors in the inset, which shows a very small difference between them, especially at several potential topological errors that are indicated with circles. To evaluate the completeness of the reconstructions, we measured their path length, which was reported as the proportion of the length to the gold standard. To quantify the accuracy of automated reconstructions, we compared them to the gold standards by using the DIADEM metric [48]. Let G be the number of nodes in the gold standard, T be the number of nodes in our reconstruction, M be the missing nodes (false negatives) reported by the DIADEM metric, and E be the extra nodes (false positive) in test reconstruction, we define the recall and precision as
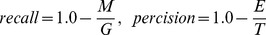



The measurements are shown in **Figure 5B**. Specifically, the path length and DIADEM score are approximately 0.95±0.03 (mean ± SD) and 0.81±0.05, respectively, which indicate that the quality of the reconstructions is generally high. In these automated reconstructions, typical errors such as branch breakings and topological connection mistakes could be easily proofread and corrected in the editing mode of flNeuronTool, which requires approximately 5∼10 minutes of user intervention per stack.

**Figure 6:**
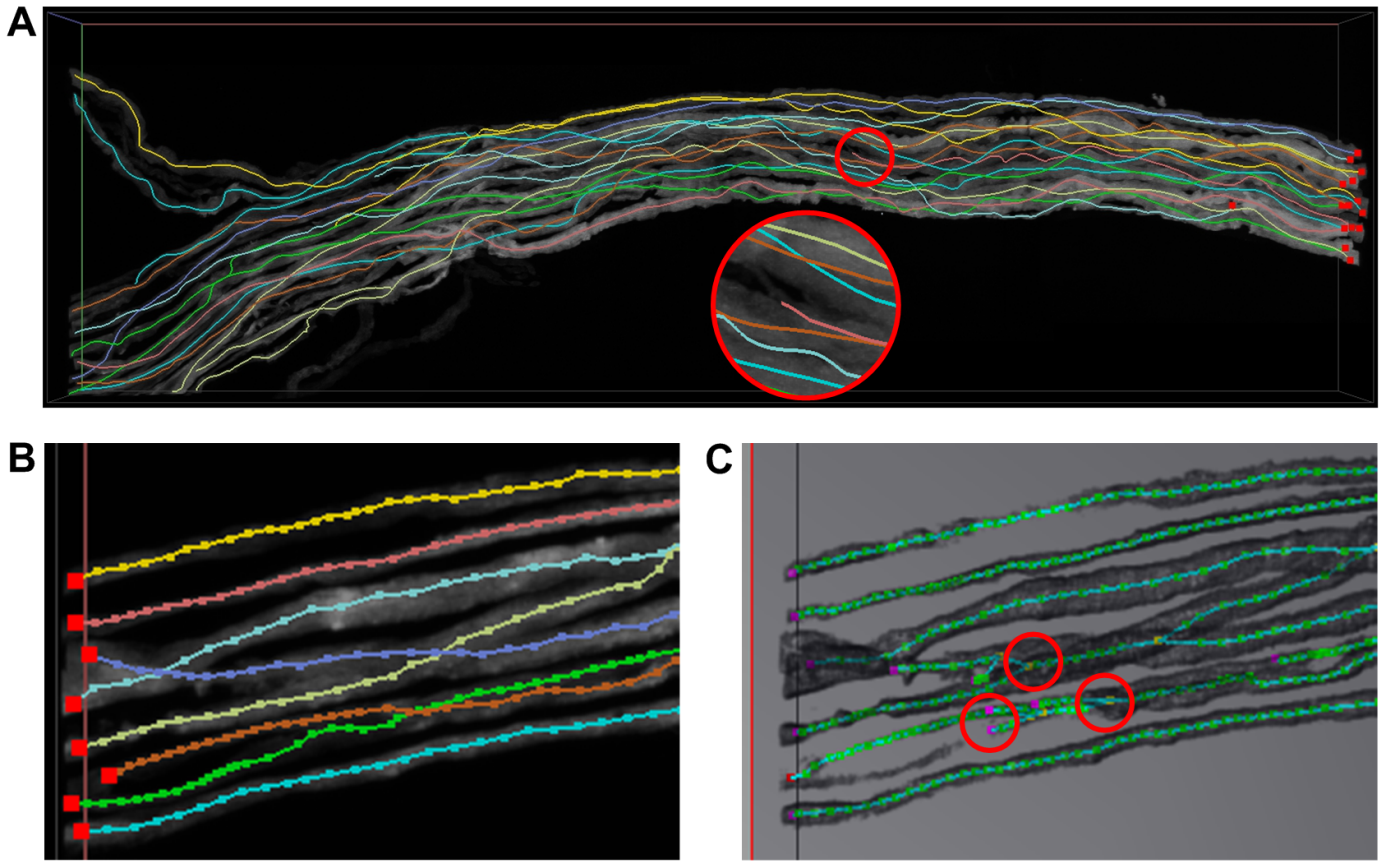
Evaluation with the DIADEM dataset NM subset 2. (A) An example of 4 image stacks (voxel size 1851×632×125) and the reconstruction, in which an error is circled. (B) An image stack (voxel size 512×512×57) and the reconstruction from flNeuronTool, which is an acceptable result without additional manual editing. (C) The automated reconstruction of the same stack from NeuronStudio, in which potential errors are circled.

The second test was run on dataset NM subset 2. As an example, we reconstructed a piece of volumetric data of 4 image stacks that have been integrated, in which 15 axon fibers start from one of the stacks and continue through the remaining stacks, as shown in **Figure 6A**. It should be noted that we could not reconstruct the entirety of the axon fibers at a high confidence level because of the absence of appropriate pre-processing. Focusing on the local detail, potential errors in the reconstruction include branch breaking from the high unevenness of the voxel intensity, one of which is indicated with a circle. Because of the differences in data dimensionality and tracing root, the DIADEM metric could not be used as expected to evaluate the quality of the reconstruction. Instead, we reconstructed a single image stack, and compared the result with the reconstruction from NeuronStudio. As shown in **Figure 6B**, flNeuronTool can create an acceptable automated reconstruction without additional manual editing. In contrast, as shown in **Figure 6C**, in the reconstruction from NeuronStudio, there are a few errors such as branch breaking or steal that are marked with circles. One possible reason of these differences is that the hemisphere sampling is more or less equivalent to smooth the original image, which could suppress the influence of individual voxels. Indeed, NeuronStudio could create a high-quality reconstruction with appropriate parameters or a small amount of manual editing. However, in our experience, the proofread in 2D projection planes that is adopted by NeuronStudio is not trivial, while the 3D interactive editing adopted by flNeuronTool is more convenient.

### Application to MOST Image

We applied the proposed system to mouse brain images acquired with the MOST system to reconstruct single neurons and local neural circuits. We used two datasets, which are from a green fluorescence labeled [40], [41] and a modified Golgi-Cox stained [38], [39] whole mouse brain and provide micron-resolution tomography images at the neurite level. For these original images, pre-processing is necessary, which strongly depends on the details of the neuron labeling. We applied flNeuronTool to pre-processed datasets to reconstruct a few single neurons and local neural circuits.

**Figure 7:**
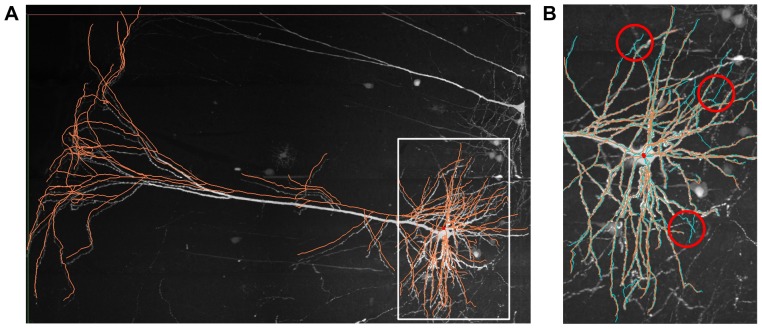
Application the proposed system to MOST fluorescence images. (A) An example of the original images (voxel size 1728×1088×176) and the reconstruction of a pyramidal neuron. (B) Detailed comparison of the reconstruction (orange) with the reference reconstruction from Amira (cyan), in which potential errors are circled (red). The proportion of total length and number of bifurcation of the reconstruction to the reference are 0.96 and 0.94, respectively.

The first subset of images was labeled using green fluorescence, which has a 3D resolution of 0.5 µm×0.5 µm×2.0 µm. An example for the reconstruction of a pyramidal neuron is shown in **Figure 7A**, in which the reconstruction was slightly shifted to improve the visibility. To evaluate the accuracy of the reconstruction, we compared it with the reference reconstruction that was created using commercial software Amira (http://www.amira.com/). A part of the details of them is displayed in different colors in **Figure 7B**, and a few of the differences between them are indicated with circles. In general, the result is acceptable. Specifically, potential errors in the reconstruction such as branch contraction could be due to uneven labeling. The proportion of total length and number of bifurcation of the reconstruction to the reference are 0.96 and 0.94, respectively. The time to achieve the final reconstruction using flNeuronTool is less than 10% of the manual method, which is very attractive for large-scale neuronal image analysis.

**Figure 8:**
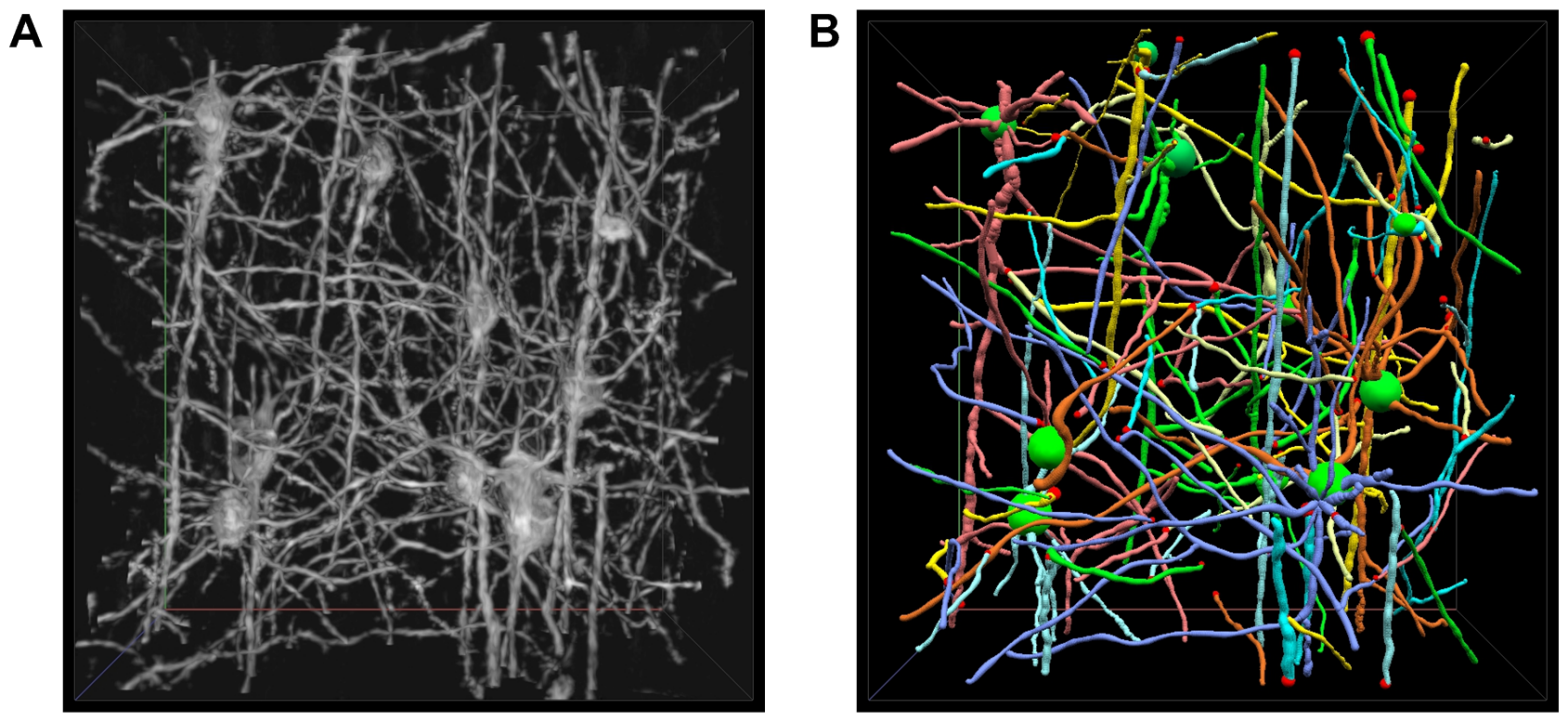
Application the proposed system to MOST Golgi images. (A) An example of the pre-processed images (voxel size 450×450×345). (B) The reconstruction that containing 10 neurons with soma (sphere) and a few separate neurite fragments.

The second subset of images was stained with the modified Golgi-Cox method, which has a 3D resolution of 0.3 µm×0.3 µm×1.0 µm. We expected to reconstruct local neural circuits from these images, because the density of neurons in this dataset is suitable. However, with our experience, it is very difficult to achieve this goal even by hand because of the complexity of the neuronal morphologies and local connections and the uneven staining. These images exhibit variable contrast, and there is frequent loss in the continuity of neuron. Hence, the best strategy is to reconstruct neuron only when the confidence level is relatively high. An example of the pre-processed images is shown in **Figure 8A**. The reconstruction from flNeuronTool is shown in **Figure 8B**, which contains 10 neurons, starting from the somas in the same volumetric subset, as well as 73 separate neurite fragments that have a length of more than 50 voxels. To illustrate the completeness of the neuronal morphologies, we used a sphere to represent a soma location. It is reasonable that the reconstruction contains a few errors, although we have spent approximately an hour on proofreading and correcting them.

## Discussion

We presented an innovative method for tracing and reconstruction of 3D neuronal morphology from light microscopy images. We validated and evaluated the proposed method using synthetic data and the DIADEM datasets, and applied the system to MOST images. The main contribution of this paper is the augmented rayburst sampling algorithm. The original rayburst sampling has been extended to a marching fashion, which is capable of using only local information to perform neurite centerline extraction, branch detection, centroid refinement and radius estimation. The advantages of the method are summarized, as follows. First, all the tracing and reconstruction steps are achieved in a single step, in which the centerline extraction or the extra radius estimation is unnecessary. Second, this method only needs to sample a part of points rather than processing all neuronal voxels one by one. Consequently, the improvement of the reconstruction speed becomes possible, while the influence of random noises is reasonably suppressed so that appropriate computational accuracy could be guaranteed. Third, the sampling is only related to the local radius but not depend on the absolute size of the structural structure, thus, it has the potential to deal with images of different spatial resolutions.

It is worth mentioning that we implemented the method in the freeware system flNeuronTool. The system incorporates automatic tracing and manual editing of neuron reconstruction into a cooperative 3D interactive visualization-assisted environment, which is a powerful tool for analysis of complex neuronal images. Experimental results showed that it provides an effective means of minimizing the human effort that is required to improve the quality of the final reconstruction. In general, the system can be faster than manual reconstruction and be more accurate than fully automatic tracing.

One limitation of the proposed method is its poor performance on beaded neurites. For these images, we think that those graph model-based algorithms may be a better choice [21]. We also found that it would be unreliable or unnecessary to reconstruct the exact morphology for some very fine dendrites which is about one voxel wide in light microscopy images. In this case, the actual application may only need to trace the centerline of neurites, and those snake-based algorithms may be more appropriate [28]. Besides, some computationally expensive but more robust algorithms could be resorted to. For example, it has been shown that the optimal path is useful for automated branch mergers or segment joins [30]. In addition, for a real large-scale dataset, such as the MOST Golgi images that are stained highly unevenly and exhibit variable contrast, a practical pre-processing platform that produces more high quality images for automated tracing and reconstruction is appreciated.

In summary, the proposed system provides an efficient means for the rapid reconstruction of 3D neuronal morphology from light microscopy images, which achieves a reasonable balance between fast speed and acceptable accuracy. This system is very promising for interactive applications of large-scale neuronal image analysis, such as the reconstruction of single neurons and local neural circuits from MOST images.

## Supporting Information

File S1 **Supporting Information for Methods, Experiments and Results.** Text S1, Sampling boundary detection along a sampling ray. Text S2, flNeuronTool: neuron reconstruction system. Text S3, Validation of the reliability of the radius estimation.
(PDF)
