# Supplementary material for: Rapid Reconstruction of 3D Neuronal Morphology from Light Microscopy Images with Augmented Rayburst Sampling
Source: PLoS One. 2013 Dec 31;8(12):e84557. doi: 10.1371/journal.pone.0084557 (PMC3877282; doi:10.1371/journal.pone.0084557)
Supplement: File S1 — Supporting Information for Methods, Experiments and Results. Text S1, Sampling boundary detection along a sampling ray. Text S2, flNeuronTool: neuron reconstruction system. Text S3, Validation of the reliability of the radius estimation. (PDF) [file pone.0084557.s001.pdf]

Rapid reconstruction of 3D neuronal morphology from light microscopy images with augmented rayburst sampling

Xing Ming, Anan Li, Jingpeng Wu, Cheng Yan, Wenxiang Ding, Hui Gong, Shaoqun Zeng, and Qian Liu*

* Corresponding author: Qian Liu. E-mail: qianliu@mail.hust.edu.cn

Supporting Information for Methods, Experiments and Results

Text S1. Sampling boundary detection along a sampling ray.

Text S2. flNeuronTool: neuron reconstruction system.

Text S3. Validation of the reliability of the radius estimation.

References

Text S1. Sampling boundary detection along a sampling ray

Those rays who reach the boundary of the local structure should be terminated early. Thus, it is important to detect the boundary along a sampling ray. In the original rayburst sampling, an intensity threshold is adopted, or a binary image is available [1]. To break those restrictions, we use a hysteresis threshold to deal with heterogeneous images, described as follows. At each time, the intensity value at endpoint P of the extended ray is obtained using tri-linear interpolation, which is defined as the sampling value, and the difference between it and the last sampling value on the same ray is defined as the sampling gradient, as shown as **Figure S1A**. In a local area around the current node, such as a cube block with a side length of 10 times the node radius, the mean of all voxels is calculated and recorded as m, while the mean of the foreground, whose voxel intensity is more than m, is recorded as h, as shown as **Figure S1B**. A ray endpoint P with a sampling value of more than h is foreground, and one with a sampling value of more than m and a sampling gradient of less than (h-m) is also regarded as foreground. The two criterions are expressed as

$$\mathrm{val}(P_i) \geq h$$

$$\mathrm{val}(P_i) \geq m, \left\| \mathrm{val}(P_i) - \mathrm{val}(P_{i-1}) \right\| \leq h - m$$

where the function val calculates the sampling value at a given point.

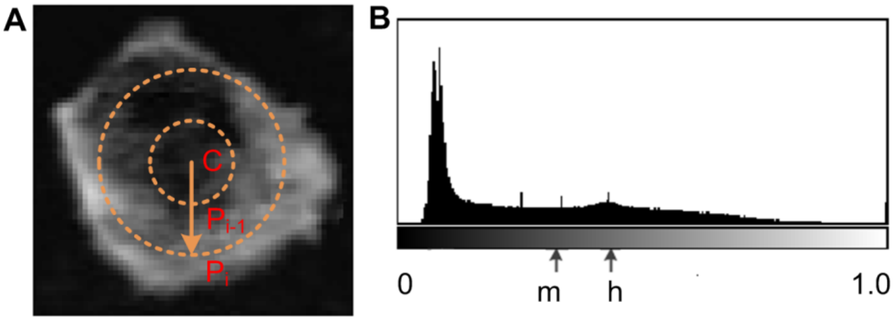

Figure S1. Detection the boundary of the structure along a sampling ray. (A) 2D illustration of the sampling value and gradient along a sampling ray. (B) Histogram of local area used to detect threshold parameters.

Text S2. flNeuronTool: neuron reconstruction system

The freeware system flNeuronTool that implemented the proposed method was built in C++ with a GUI based on OpenGL (http://www.opengl.org/) and FLTK (http://www.fltk.org/). It potentially supports multiple platforms. This system is partly inspired by Vaa3D [2] and FARSIGHT trace editor [3] and selectively combines their most important features, which allows users to reconstruct and proofread multiple neuronal morphologies in a cooperative 3D interactive visualization-assisted environment, as shown as **Figure S2**. The source code and binaries are freely available at http://sourceforge.net/projects/flneurontool/. The current version includes basic features, as follows:

- File: Loading and exporting an image stack in the TIFF format, single or multiple neuron reconstructions in the SWC format.
- Visualization: The image stack is visualization using GPU based volume rendering, on which multiple neuron reconstructions are conveniently displayed overlaid in different colors and styles. User interaction and direct object pick in 3D are provided.
- Tracing: Automatic tracing and reconstruction of neuronal morphology based on the algorithm described above is the core of the system.
- Editor: Errors in the automated reconstruction can be corrected in manual editing mode. Individual tree segments can be added, rotated, jointed, split and removed. A few batch operations, such as radius and branch fix ups, are also permitted.
- Analysis: A few statistic features of reconstructions can be counted.
- Thread: The system supports multi-threading processing, by which user operations can be quickly responded while performing time-consuming calculation.

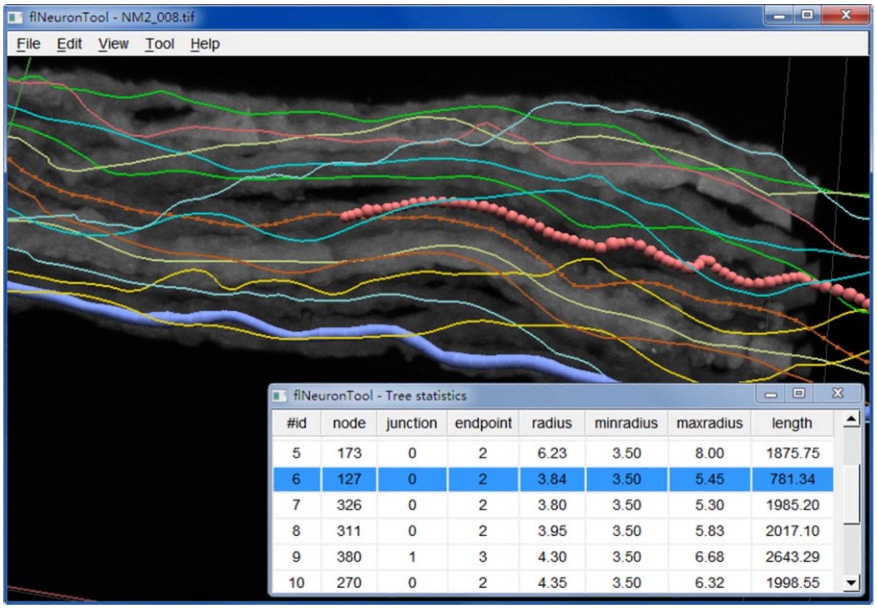

Figure S2. GUI of flNeuronTool. Multiple reconstructions are displayed overlaid on the voxel data in different colors and styles. The table at right shows the statistical information of completed reconstructions.

Text S3. Validation of the reliability of the radius estimation

We validate the reliability of the radius estimation, because the local radius is the basis of the hemispherical sampling that is used to predict centerline and branch. We use synthetic data that has helix-like structure and varying width [4]. The image and the automated reconstruction from flNeuronTool are shown in **Figure S3A**. As comparison, we reconstruct the same structure using NeuronStudio [5] and OpenSnake [6], and analyze the change of the local radius along the z axis, as shown in **Figure S3B**. In the results, changes of the radius estimation are highly consistent for different reconstructions, which show that all of the methods are acceptable. However, for some positions, the radius value from NeuronStudio appears bigger than the two others. The possible reason is that NeuronStudio estimates radius on the axial cross-sectional plane, which could cause deviation when the structure is oblique crossing the plane. Potentially, the radius estimation that is available in 3D has higher credibility.

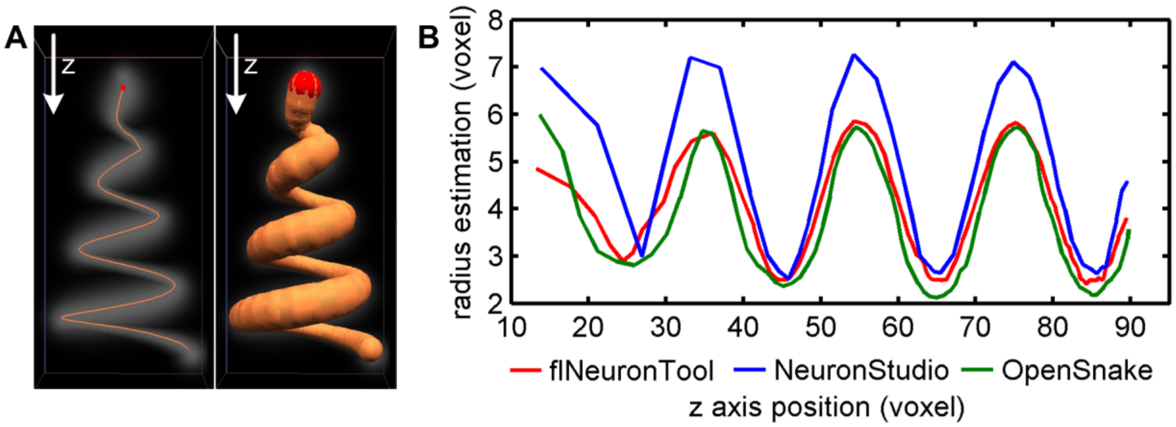

Figure S3. Validate the reliability of the radius estimation on helix-like data. (A) The image (voxel size 50×50×100) and automatic reconstruction from flNeuronTool are displayed in two different styles. (B) The change of the local radius along the z axis for different reconstructions from flNeuronTool, NeuronStudio and OpenSnake, respectively.

References

1. Rodriguez A, Ehlenberger DB, Hof PR, Wearne SL (2006) Rayburst sampling, an algorithm for automated three-dimensional shape analysis from laser scanning microscopy images. Nature Protocols 1: 2152-2161.
2. Peng H, Ruan Z, Long F, Simpson JH, Myers EW (2010) V3D enables real-time 3D visualization and quantitative analysis of large-scale biological image data sets. Nature Biotechnology 28: 348-353.
3. Luisi J, Narayanaswamy A, Galbreath Z, Roysam B (2011) The FARSIGHT trace editor: an open source tool for 3-D inspection and efficient pattern analysis aided editing of automated neuronal reconstructions. Neuroinformatics 9: 305-315.
4. Krissian K, Malandain G, Ayache N, Vaillant R, Trousset Y (2000) Model-based detection of tubular structures in 3D images. Computer Vision and Image Understanding 80: 130-171.
5. Rodriguez A, Ehlenberger DB, Hof PR, Wearne SL (2009) Three-dimensional neuron tracing by voxel scooping. Journal of Neuroscience Methods 184: 169-175.
6. Wang Y, Narayanaswamy A, Tsai C-L, Roysam B (2011) A broadly applicable 3-D neuron tracing method based on open-curve snake. Neuroinformatics 9: 193-217.
